# Establishment of a standardized dietary model for nanoparticles oral exposure studies

**DOI:** 10.1002/fsn3.2112

**Published:** 2021-01-15

**Authors:** Yan Li, Kun Jiang, Hui Cao, Min Yuan, Tai Ye, Fei Xu

**Affiliations:** ^1^ School of Medical Instrument and Food Engineering Shanghai Engineering Research Center for Food Rapid Detection University of Shanghai for Science and Technology Shanghai China

**Keywords:** food matrixes, nanoparticles, stability, standardized dietary model

## Abstract

Food matrices could affect the physicochemical properties of nanoparticles (NPs) and define the biological effects of NPs via oral exposure compared with the pristine NPs. We established a standardized dietary model based on Chinese dietary reference intakes and Chinese dietary guidelines to mimic the exposure of NPs in real life and to evaluate further the biological effect and toxicity of NPs via oral exposure compared with current models. The standardized dietary model prepared from the primary emulsion was dried into powder using spray drying compared with commercial food powder and then was reconstituted compared with the fresh sample. The average particle size (295.59 nm), potential (−23.78 mV), viscosity (0.04 pa s), and colors (L^*^, a^*^, b^*^ = 84.13, −0.116, 8.908) were measured and characterized of the fresh sample. The flowability (repose angle = 37.28° and slide angle = 36.75°), moisture (2.68%), colors (L^*^, a^*^, b^*^ = 94.16, −0.27, 3.01), and bulk density (0.45 g/ml) were compared with commercial food powder. The size (310.75 nm), potential (−23.98 mV), and viscosity (0.04 pa s) of reconstituted model were similar to the fresh sample. Results demonstrated that the model was satisfy the characterizations of easy to fabrication, good stability, small particle size, narrow particle size distribution, strong practicability, and good reproducibility similar to most physiological food state and will be used to evaluate NPs’ safety.

## Graphical abstract

(a) The standardized dietary models have the characteristics of easy to formation, small particle size and narrow particle size distribution, high uniformity, and good stability; (b) the powdered standardized diet model has a moisture content, good flowability, and strong white appearance, which is helpful for practical production and application, and was reconstituted similar to the fresh sample of properties; (c) the model will be used to evaluate NPs’ safety.

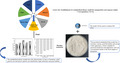

## 1 INTRODUCTION

Recently, nanotechnology is one of the fastest growing areas of science, which involves the development, characterization, and application of nanoparticles (NPs). More and more NPs exhibiting specific and novel physicochemical properties have been produced and applied in food and pharmaceutical industry in production, packaging, sensors, nutrient delivery systems, and food additives to improve products properties including visual presentation, products quality, shelf life, safety, and nutritional absorption (Chen et al., 2015; McClements & Xiao, 2017). For example, titanium dioxide NPs are used as a popular food additive in various food products such as gum, candies, puddings, cheeses, sauces, skimmed milk, ice creams, pastries, dressings, and certain powdered food in order to enhance the white color, brightness and improve the flavors of food (Dudefoi et al., 2017; Li, Yang, et al., 2018). Silica NPs are added into certain powdered food products such as salts, icing sugar, spices, dried milk, and dry mixes as anti‐caking agents in order to prevent poor flow or “caking.” Silver NPs are extensively used in food packaging materials owing to broad‐spectrum antimicrobial activity against microorganisms (Winkler et al., 2016).

The fast‐growing applications of NPs in food and pharmaceutical industry enhance the oral exposure possibility. NPs added in food and pharmaceutical products pass through the human gastrointestinal tract (GIT) upon ingestion, which may induce the physicochemical transformations and redefine NPs’ toxic and health effects, consequently (Mittag et al., 2019; Orr et al., 2019; Tang et al., 2018). Some recently studied also evidenced the acute toxicity and biodistribution of different sized copper NPs in rats after oral administration (Cao et al., 2016; PRC, 2017a). Extensive studies compared the NPs incorporated food components with pristine NPs via oral exposure both in vivo and in vitro, and found that the interaction between NPs and food components could be the main reason of the NPs’ physicochemical properties transformations (Ahamed et al., 2019; Jiang et al., 2019; Jo et al., 2016). Many recently investigations assessing the toxicological impacts of ingested NPs have considered the interactions between NPs and food components. The above‐mentioned various food components considered mainly included typical food ingredients, such as proteins, carbohydrates and minerals, and lipids, contain a variety of molecular and colloidal species that can induce different NPs physicochemical transformations such as their solubility, surface composition, electrical charge, and aggregation state (Bae et al., 2018; Chen et al., 2015; Di Silvio et al., 2016; Go et al., 2017, 2018; Jiang et al., 2016; Laloux et al., 2020; Lee et al., 2017; Li, Zhang, et al., 2018; Lichtenstein et al., 2015; Ramos et al., 2017). These studies lack methodological standardization, and results not can be reliably compared between different laboratories, so that the standardized food model is really needed for different researchers; especially, some food models to represent the typical different dietary habits should be developed. Additionally, food models for other more specialized diets including high‐fat, high‐sugar, or high‐protein diets also need to be developed.

At present, a few researchers have made good work in this field. Ensure^®^ Plus and whole milk (3.5%) both are simple food models that have been used to evaluate analyte dissolution and bioavailability or bioequivalence studies, and are similar to the HHS‐FDA recommended standard meal for the effects of drug or food. DeLoid et al. chose a simple food matrix composed of oil‐in‐water (O/W) emulsion stabilized by a protein to develop an integrated methodology for assessing food matrix and GIT effects on NPs’ biokinetics and toxicology (DeLoid et al., 2017; Jantratid et al., 2008). Based on the typical US diet, Zhang et al. developed a standardized food model used to assess TiO_2_ NPs, the results showing the presence of food matrix mitigated the potential cytotoxicity of TiO_2_ NPs > 5‐fold, which demonstrating the importance of food matrix effects (Zhang et al., 2019).

In this study, a standardized dietary model based on Chinese Dietary Reference Intakes and Chinese Dietary Guidelines from the average for men and women of 18 years or older was developed (PRC, 2017a). We used different methods of ultrasonic homogenization and high‐pressure homogenization in processing, and examined the various indexes of average particle size, polydispersity index (PDI), surface charge, viscosity, microstructure, and stability. The physical properties of powdered standard dietary model using spray drying were also discussed compared with whole milk powder in order to facilitate the practical application of the model. Moreover, the reconstituted standard dietary model properties (particle size, surface charge) were also measured compared with the fresh samples. Overall, this developed standardized dietary model could be used to better imitate intake of NPs in real life, to better evaluate NPs’ biological effects and toxicity, and to better perform an accurate risk assessment via oral exposure in vitro and vivo. In addition to this, many other studies, such as the bioavailability of pharmaceuticals, nutraceuticals, or pesticides, also should take the standardized dietary model into account.

## 2 MATERIALS AND METHODS

### 2.1 Materials

Sodium caseinate, pectin, sucrose, and sodium chloride were purchased from Macklin Biochemical Co. and a local food supplier, respectively. Na_2_HPO_4_, NaH_2_PO_4_, Nile red, and fluorescein thiocyanate isomer I (FITC) were purchased from Sigma‐Aldrich. Modified starch and corn oil were obtained from a local food supplier. All chemicals were analytical grade. Double‐distilled water was used in all solutions.

### 2.2 Emulsion preparation and characterization

#### 2.2.1 Emulsion preparation

The preparation and characterization of emulsions were carried out using procedures described previously (Corzo‐Martinez et al., 2011; Hu et al., 2017; Loi et al., 2019; Perugini et al., 2018; Troncoso et al., 2012; Zhang et al., 2015a, 2015b). Sodium caseinate (1%, w/w) was dissolved in phosphate buffer solution (10 mM, pH 7) that was continually stirred using a magnetic stirrer until complete dissolution at room temperature. Sodium caseinate solution was then filtered to remove undissolved particles (the final protein concentration would not be significantly changed). Corn oil (3.21%, w/w) was then added to sodium caseinate solution with magnetic stirring for 10 min and then homogenized using high‐speed mixer at 16,000 r/min for 2 min (IKA Instruments) to prepare a coarse emulsion. The coarse emulsion was then, respectively, passed through an ultrasonic cell disruptor (Sonics Instruments) and a high‐pressure homogenizer (APV Instruments) to create an initial fine emulsion. The operational pressure of high‐pressure homogenizer was carried out at 6,000, 9,000, and 12,000 psi, respectively, for three passes. The operational power of ultrasonic cell disruptor comprised a tapered microtip (6 mm diameter) was carried out at 400, 500, and 600 W, respectively, for 2‐s interval and 6‐min total time.

#### 2.2.2 Size and PDI of emulsions

The average particle size and PDI of the fine emulsions were determined by Dynamic Light Scattering (DLS) using a NanoBrook 173Plus (Brookhaven Instruments). The refractive indices were 1.47 for oil and 1.33 for water. All measurements were performed at 25°C.

#### 2.2.3 Zeta potential of emulsions

Zeta potential of the fine emulsions was determined through the electrophoretic mobility measurements of dispersed phase by laser Doppler velocimetry using a Zetasizer Nano S90 (Malvern Instruments). All measurements were performed at 25°C.

#### 2.2.4 Viscosity of emulsions

Viscosity of the fine emulsions was measured using a Discovery HR‐3 rheometer (TA instruments, US) equipped with a conical plate with 60 mm diameter. The fine emulsions were carefully placed on the surface of conical plate, and the upper plate was then down‐regulated to reach 1‐mm gap distance. The fixed shear rate range was 10 to 100 s^−1^ at 25°C.

#### 2.2.5 Microstructure of emulsions

Two milliliter of samples was mixed with 0.1 ml Nile Red solution (1 mg/ml ethanol) and 0.1 ml FITC solution (10 mg/ml dimethyl sulfoxide) to dye oil and protein for 30 min, respectively. The microstructure was observed using fluorescence microscope (Thermo Scientific).

#### 2.2.6 Stability of emulsions

The stability of all fine emulsions was stored at 4 and 25°C for 24 or 48 hr in dark, respectively, and was then subjected to analysis on size, PDI, and zeta potential. Meanwhile, the cream layer of emulsions at each time and temperature was also observed.

### 2.3 Standardized dietary model preparation and characterization

#### 2.3.1 Standardized dietary model preparation

The formula of standardized dietary model was based on Chinese dietary reference intakes and Chinese dietary guidelines (PRC, 2017a). The nutrient formula of the standardized dietary model established in this study is summarized in Table 1.

**TABLE 1:** The nutrient formula of the standard dietary model based on Chinese dietary reference intakes and Chinese dietary guidelines (PRC, 2017a)

Dietary components	Level (g/100 g)
Protein (sodium caseinate)	2.89 g/100 g
Fat (refined corn oil)	3.21 g/100 g
Dietary fiber (pectin)	1.32 g/100 g
Starch (modified corn starch)	13.8 g/100 g
Sugar (sucrose)	1.44 g/100 g
Minerals (sodium chloride)	0.37 g/100 g

Additional sodium caseinate was slowly added into the optimized initial emulsion at ambient temperature with continual stirring to ensure full dissolution (final protein concentration = 2.89% w/w; final fat concentration = 3.21% w/w). The other dietary components were sequentially added to reach the final concentration: pectin (1.32% w/w), starch (13.8% w/w), sucrose (1.44% w/w), and sodium chloride (0.37% w/w).

#### 2.3.2 Characterization of standardized dietary model

The samples of after sequentially adding each of the components were collected and used to characterize the size, PDI, zeta potential, viscosity, microstructure, optical property, and stability. The methods are similar to the characterization methods of emulsions. Samples were properly diluted with phosphate buffer solution to avoid multiple scattering effects when size, PDI, and zeta potential were measured. Different from the emulsion, samples were directly observed in bright‐field microscope to research microstructure (Hu et al., 2017; Zhang et al., 2015a, 2015b).

The tristimulus color coordinates of the samples of after sequentially adding each of the components were measured using a ColorFlez EZ colorimeter (HunterLab Instruments) to research optical properties. The procedure was described previously (Zhang et al., 2019). L* (lightness), a* (red to green), and b* (yellow to blue) are tristimulus color coordinates.

### 2.4 Powdered standardized dietary model preparation and characterization

#### 2.4.1 Standardized dietary model powder preparation

The freshly standardized dietary model samples were dried into powder using a JYP‐1500 spray dryer (Shanghai Juyuan Machinery Co., Ltd) at an inlet temperature of 180°C, a feed flow rate of 0.35 L/hr, and a nozzle atomizer diameter of 0.75 mm, which was adopted from some previous studies with slight modifications. In this drying process, the freshly samples were continually stirred using a magnetic stirrer. The powdered samples were then collected and stored at 4°C.

#### 2.4.2 Repose angle of powder

The repose angle of standardized meal model powder and whole milk powder (Inner Mongolia Yili Industrial Group Co., Ltd) was carried out using procedures described previously (Zhang et al., 2019). A funnel was fixed vertically above a horizontally drawing with a distance (H) from the funnel outlet to the drawing. The powdered samples freely falling along the outlet direction of funnel until the top of the powder contacted the funnel outlet. The base diameter of the formed cone was 2R. The repose angle was calculated as the following formula:

Reposeangle=arctanH/R,

#### 2.4.3 Slide angle of powder

The angle of slide of standardized meal model powder and whole milk powder were performed using procedures described previously (Zhang et al., 2019). The powdered samples were evenly distributed on a rectangular horizontal glass plate with a length of L. The glass plate surface was then slowly raised until the surface powdered samples began to slide. The vertical distance from the top of the inclined glass plate to the horizontal plane was H. The slide angle was calculated as the following formula:

Slideangle=arcsinH/L,

#### 2.4.4 Bulk density of powder

The bulk density of standardized meal model powder and whole milk powder was determined using procedures described previously (Goula & Adamopoulos, 2005). The powdered samples were placed in a graduated cylinder with continual vortexing until the volume of powdered samples was constant. The bulk density was calculated as the following formula:

Bulkdensity=powderedsamplemass/volumeingraduatedcylinder,

#### 2.4.5 Moisture content of powder

The moisture content of standardized meal model powder and whole milk powder was determined using “Direct drying method” from GB 5009.3‐2016 of National Food Safety Standard (PRC, 2017b). Briefly, the weighing bottle was placed in drying oven at 101°C for 60 min and cool for 30 min before weighing. Repeat the drying until the quality difference between the before and after does not exceed ±2.0 mg (mass = *m*_3_). Then, the powder samples were placed in dry weighing bottle and weighed it until the quality difference between the two weighings does not exceed ±0.001 g (mass = *m*_1_). Finally, the weighing bottle containing the powder sample was dried at 101°C for 120 min and cool for 30 min before weighing. Repeat the drying until the quality difference between the before and after does not exceed ±2.0 mg (mass = *m*_2_). The moisture content was calculated as the following formula:

$$\text{Moisture}\;\text{content}=\left(m_{1}‐m_{2}/m_{1}‐m_{3}\right)\times 100,$$

### 2.5 Reconstituted standardized dietary model preparation and characterization

Powdered standardized dietary model (23.03 g) was dissolved in double‐distilled water (76.97 g) at ambient temperature with continual stirring to ensure full dissolution. The reconstituted standardized dietary model properties (particle size and surface charge) were also measured and compared with the fresh samples. The methods are similar to the characterization methods of emulsions. Samples were properly diluted with phosphate buffer solution to avoid multiple scattering effects.

### 2.6 Data analysis

All the results were presented as mean ± standard deviation (*SD*) of the triplicates for each prepared samples, and analyzed using the SPSS software (IBM SPSS Statistics 25). One‐way analysis of variance (ANOVA) followed by Dunnett's multiple comparison tests was used for the statistical analysis of results. Different letters in the table also indicate significant difference (*p* < .05).

## 3 RESULTS AND DISCUSSIONS

### 3.1 Characterization of emulsion

**FIGURE 1:**
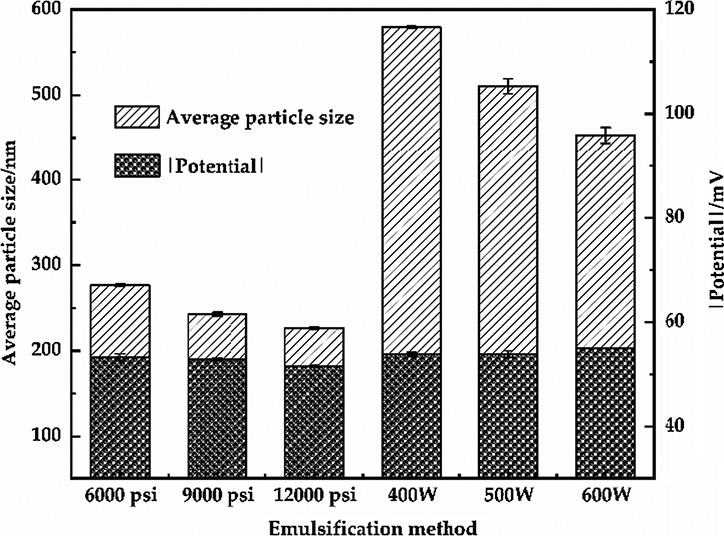
Average particle size and Zeta potential of the primary emulsions with different emulsification methods

Emulsions are usually complex systems formed by mechanical shearing of oil, water, and emulsifiers. Proteins are commonly used as emulsifier due to that they can promote the formation of O/W emulsions, maintain the dynamic stability of O/W emulsions, and control the rheological property of O/W emulsions (Dridi et al., 2018). Protein‐stabilized O/W emulsions are the foundation of many foods, such as milk, yogurt, beverages, gravy, mayonnaise, and many other foods (Loi et al., 2019; Zhang et al., 2015). Sodium caseinate, sodium salt form of casein, has great emulsification and thickening properties and has been widely used in food industry as emulsifier (Perugini et al., 2018). High‐pressure homogenization technique is the most commonly used method for emulsification of O/W emulsions, but it has the disadvantages of lower efficiency and easy energy dissipation. Compared with the traditional mechanical emulsification method, ultrasonic homogenization technique has gained extensive attention due to it can form fine and stable emulsions with relatively low energy loss (Corzo‐Martinez et al., 2011; Hu et al., 2017). In this study, high‐pressure and ultrasonic homogenization were used to prepare the primary emulsion and the emulsification stability of different emulsions were then compared and analyzed in order to provide the premise for preparing a stable and uniform standardized dietary model. Figure 1 showed that the mean particle size of the primary emulsions obtained by different emulsification methods were significant difference (*p* < .05). A significantly decreased the average particle size of samples by high‐pressure homogenization in comparison with using ultrasonic (*p* < .05). The emulsion by 12,000 psi using high‐pressure homogenization has the smallest average particle size, that is, the great stability. Moreover, except for 600 W emulsified emulsion, there was no significant difference (*p > .05*) between the emulsions by both techniques in zeta potential (absolute value). The absolute value of the potential of all emulsions was above 50, corresponding to the results of PDI (all <0.2), which indicated a narrow size and great stability of all emulsions. These results suggested that the emulsions by high‐pressure homogenization were more uniform and stable compared to the emulsions by ultrasonic.

**FIGURE 2:**
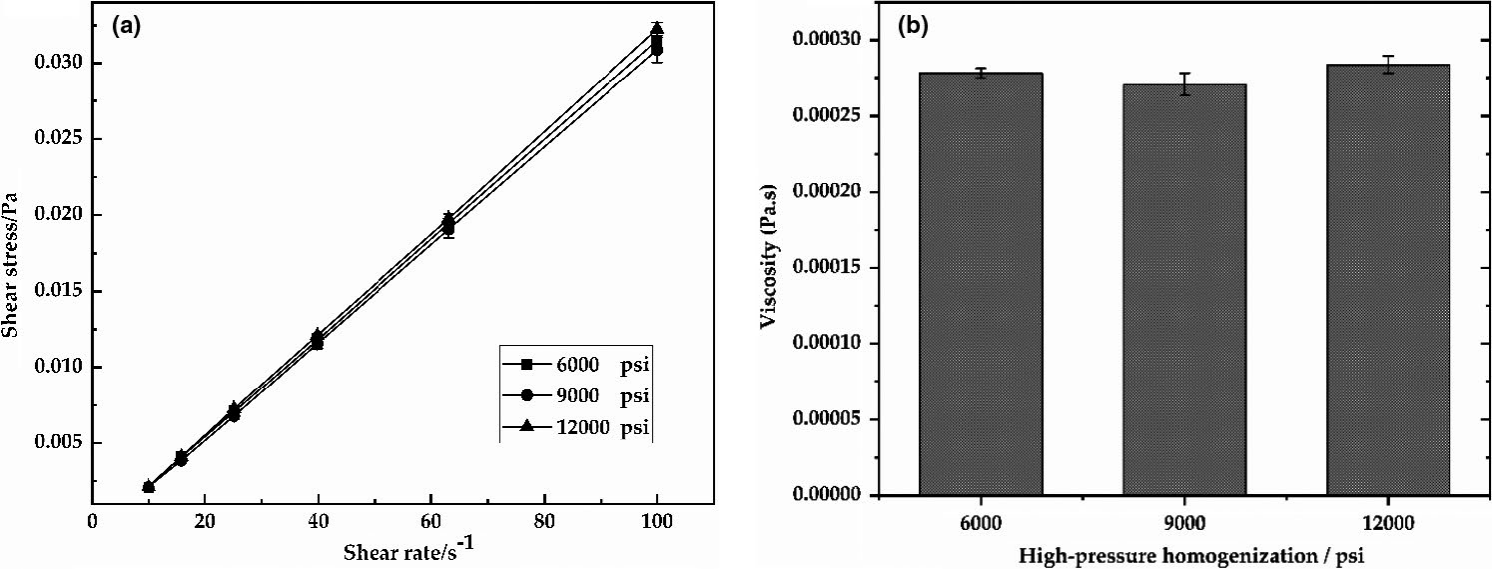
Shear stress versus shear rate (10–100s^−1^) profiles (a) and viscosity (b) of the primary emulsions with different emulsification pressures (high‐pressure homogenization)

**FIGURE 3:**
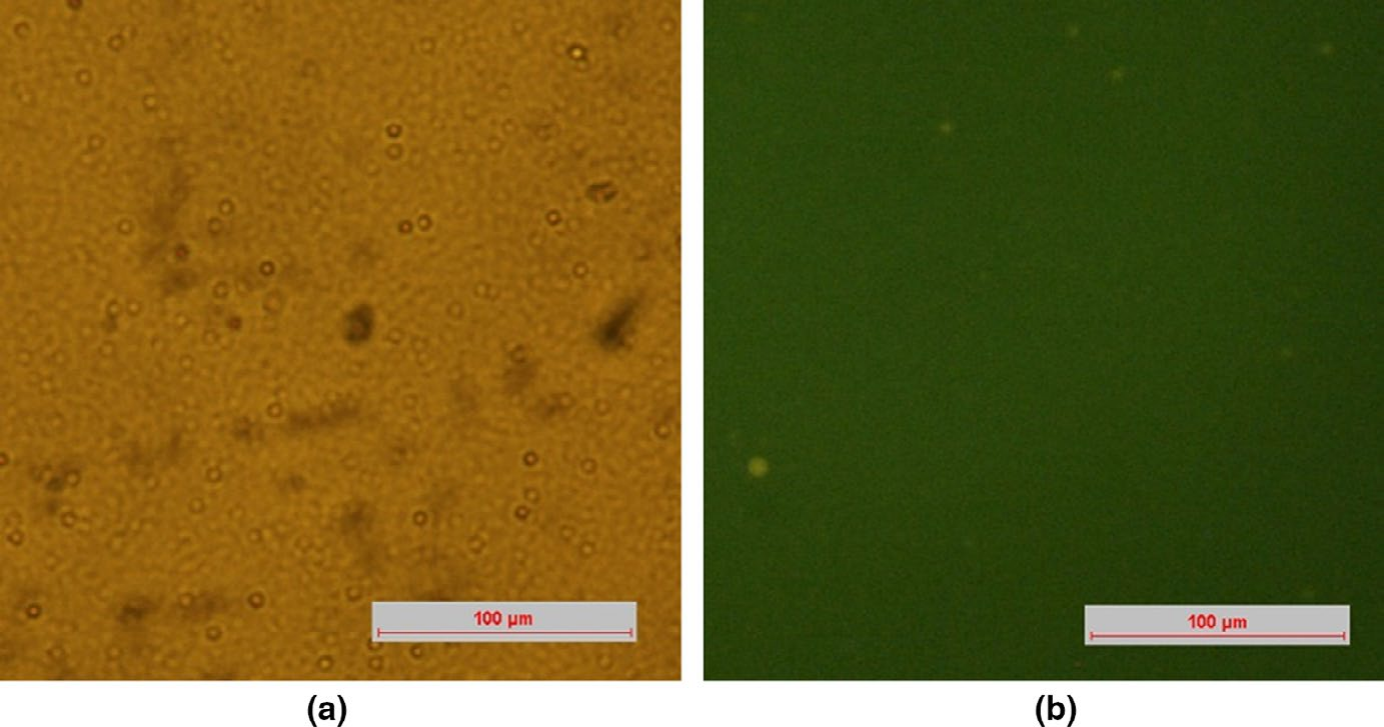
Optical microscope images of the primary emulsion (12,000 psi, high‐pressure homogenization): (a) bright‐field image; (b) fluorescent image

Generally, emulsion with higher of the viscosity has greater resistance and better stability. Figure 2a showed a linear relationship between shear stress and shear rate, which suggested that the flow behavior of a Newtonian fluid was presented. Figure 2b showed that the viscosity of the emulsions by high‐pressure homogenization in 6,000, 9,000, and 12,000 psi (0.00027, 0.00027, and 0.00028 Pa s) was no significant difference (*p* > .05). The emulsion by 12,000 psi had slightly higher viscosity and better stability, corresponding to the results in Figure 2. Moreover, Figure 3 showed the microstructure of emulsions using high‐pressure homogenization in 12,000 psi. The distribution of emulsion droplets was uniform and not observed aggregation by microscope images, which was in agreement with the value of PDI.

**FIGURE 4:**
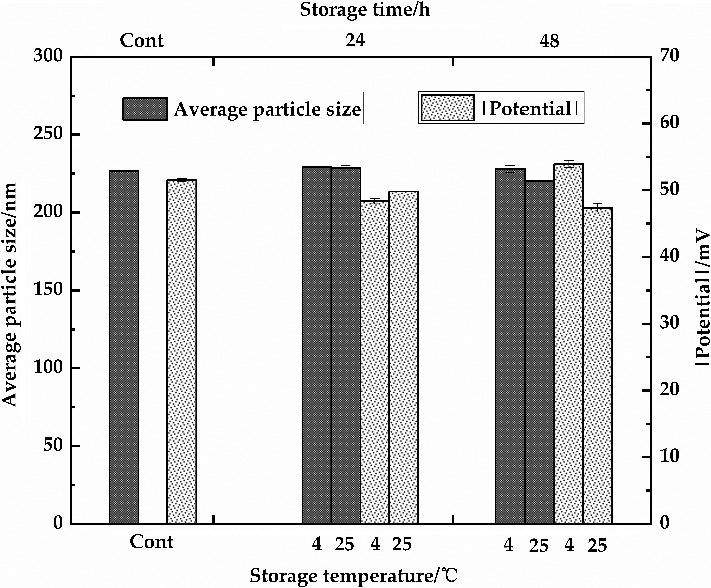
Analysis of stability of the primary emulsions (12,000 psi, high‐pressure homogenization)

Figure 4 showed that the average particle size and potential absolute value of emulsions by high‐pressure homogenization in 12,000 psi were unchanged (*p* > .05) and slightly decreased observing under 50 (*p* < .05) after storage of 24 hr or 48 hr at 4 and 25°C, indicated the great stability of the emulsion. Compared with the storage at 25°C, the absolute value of potential was no significantly different at 4°C for 24 hr or 48 hr (*p* > .05), indicated the good storage temperature at 4°C. These results suggested that the properties of the primary emulsions by high‐pressure homogenization in 12,000 psi are better and following used. The stability of emulsions is important parameters of the food products. High‐pressure and ultrasonic homogenization are most used to prepare food grade nano‐emulsions (Anwar et al., 2020; Calligaris et al., 2018; Li et al., 2017; Li & Xiang, 2019). In contrary, a better performance of ultrasound was observed in food and beverage industries (Li & Xiang, 2019), which depend to the composition of emulsion.

### 3.2 Characterization of standardized dietary model

Various dietary ingredients were added to the above optimized emulsion obtained the standardized dietary model as the following order: sodium caseinate (2.89%, w/w), pectin (1.32%, w/w), starch (13.8%, w/w), sucrose (1.44%, w/w), and sodium chloride (0.37%, w/w).

**FIGURE 5:**
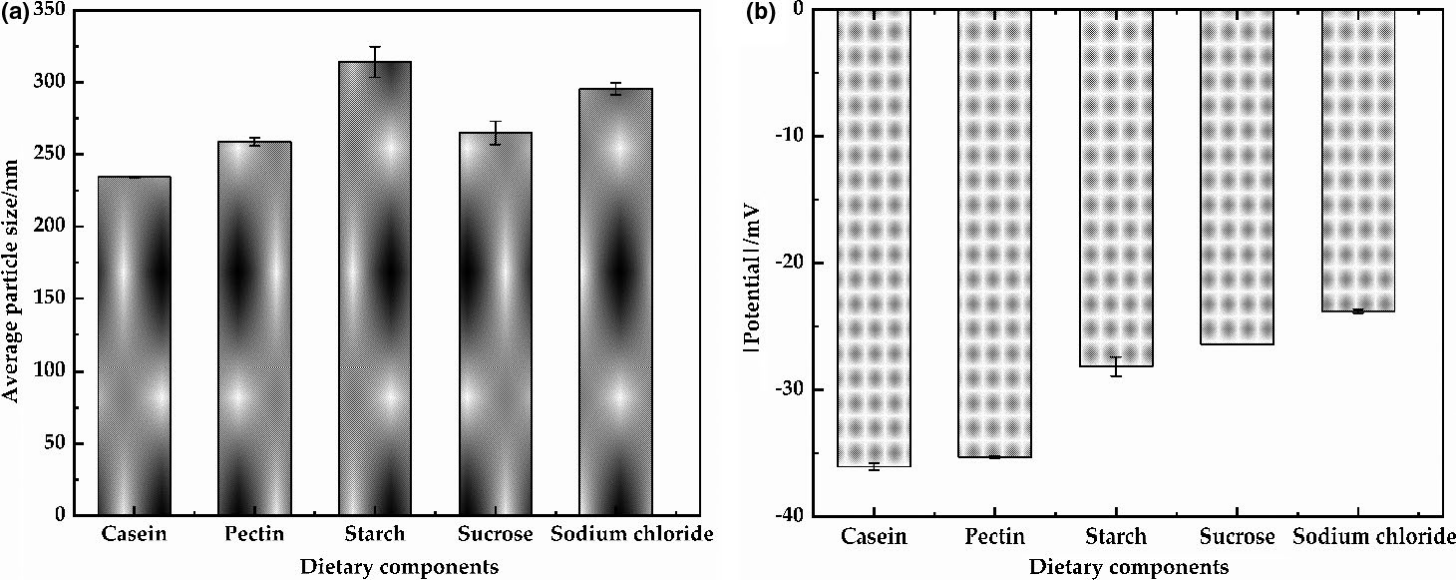
Average particle size (a) and Zeta potential (b) of the models after sequentially adding each of the dietary components

The average particle size, zeta potential, viscosity, color, and microstructure of the systems after sequentially adding each of the dietary components were characterized. Figure 5 showed that the particle size and zeta potential (absolute value) of the each system after adding a dietary component were little change and tend to be stable, respectively, suggesting a relatively stable aggregation state of the standardized dietary model. Sodium caseinate and pectin molecules are negatively charged (pH 7) in the model, which leads to the strong electrostatic repulsion among molecules (Surh et al., 2006). Starch could more effective decreased the stability of the system through increasing the average particle size and decreasing the absolute zeta potential value than other ingredients. Starch and sucrose are neutral molecules, which would not affect the electrostatic interaction of the system. However, starch could more effective affect the stability of the model due to the large addition amounts of large hydrophilic molecule. As slightly increased and decreased the average particle size and the absolute zeta potential value of the samples after adding sodium chloride in comparison with other samples, respectively, which might be attributed to the electrostatic shielding effect (Babenko et al., 2015). It is worth noting that the sample must be diluted to avoid multiple scattering effects before size and potential analysis. Therefore, these results can only be used as a rough estimate of the actual particle size and potential of the model.

**FIGURE 6:**
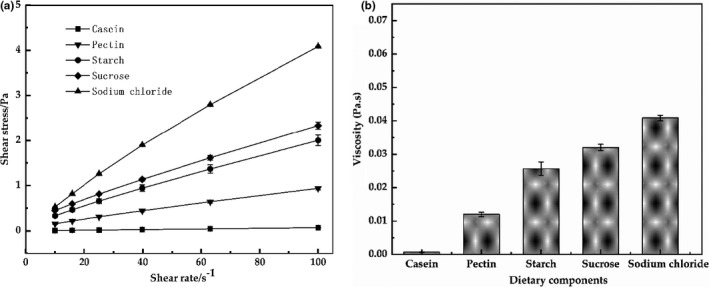
Shear stress versus shear rate (10–100s^−1^) profiles (a) and viscosity (b) of the models after sequentially adding each of the dietary components

**FIGURE 7:**
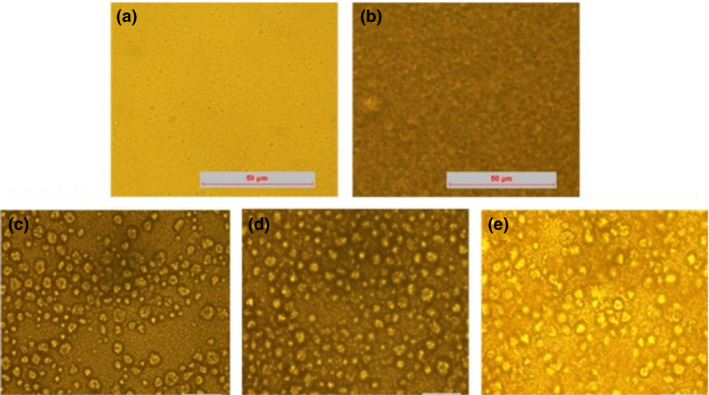
Optical microscope images of the models after sequentially adding each of the dietary components: (a) Casein; (b) Pectin; (c) Starch; (d) Sucrose; (e) Sodium chloride

The rheological property of the models after sequentially adding each dietary component is shown in Figure 6. The viscosity of the model was lower after adding casein, and then, the viscosity of the models sequentially increased after adding other components sequentially, indicating the retarded droplet movement in the systems. Pectin and starch, large hydrophilic molecules, could lead to an increased viscosity of the system by reducing the fluidity (Seo et al., 2018). Moreover, the presence of sugar and salt might change the interaction of molecules and colloids in the system. Moreover, Figure 7 shows that the microstructure of the models after sequentially adding each dietary component. The aggregation state of the systems was significantly increased and tend to be stable by microscope images, however, which were not in agreement with the results of size by DLS due to the fact that the nonadsorbing pectin promoted O/W emulsion flocculation and hydrophilic starch increased the viscosity of the systems, while the diluted models promoted flocculation dissociation due to the reduced concentration of pectin and starch (Guzey & McClements, 2006; Zhang et al., 2019).

**FIGURE 8:**
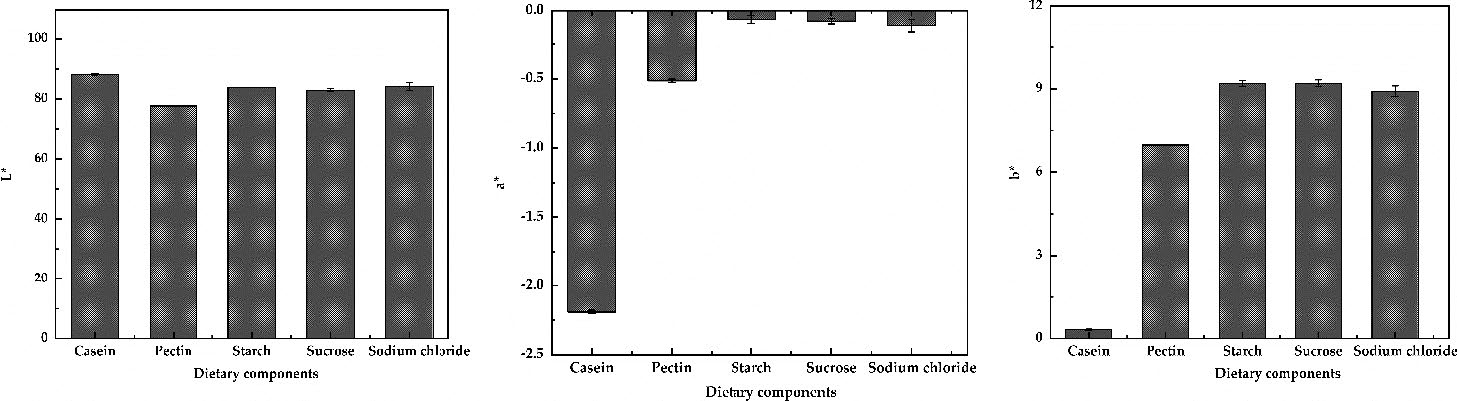
Optical properties (L^*^, a^*^, b^*^) of the models after sequentially adding each of the dietary components

**FIGURE 9:**
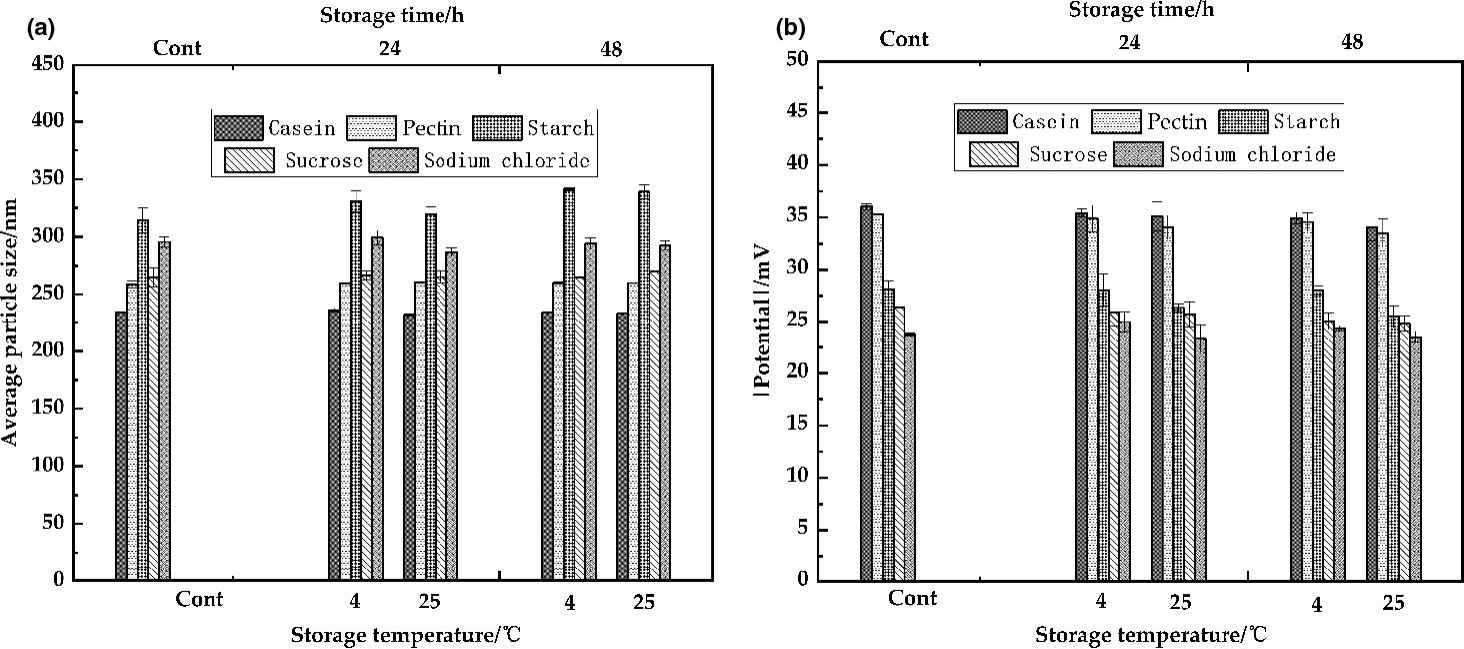
Analysis of stability of the standardized dietary model. (a) average particle size; (b) absolute potential value

The optical properties of the system can indirectly reflect its stability due to the influence of droplet concentration and aggregation state (Chung et al., 2014). The L* and absolute a* value were decreased, and the b* value was increased after adding pectin, indicating that the brightness of the system decreased and turned yellow. L*, a*, and b* were not statistically significantly different after adding starch, sucrose, and sodium chloride (*p* > .05), indicating a high stability of the systems as shown in Figure 8. The standardized dietary model was stored at 4 and 25°C for 24 hr or 48 hr, which was not significantly different in average particle size (*p* > .05), suggesting the model was relatively stable as shown in Figure 9a. Meanwhile, Figure 9b shows that the absolute potential value of the model did not change significantly (*p* > .05) after 24 hr or 48 hr at 4°C, which indicated the good storage temperature of the model at 4°C. The standardized dietary models were stable to creaming throughout 48 hr at 4 and 25°C; however, the creaming of the fresh samples was not observed due to the increase of viscosity and the formation of three‐dimensional network structure of flocculated oil droplets (Chung et al., 2014). Taken together, the standardized dietary models have the characteristics of easy to formation, small particle size and narrow particle size distribution, high uniformity, and good stability.

### 3.3 Characterization of powdered standardized dietary model

Spray drying, an efficient, economic, and flexible drying technique, is increasingly used in dairy, food, chemical, and biopharmaceutical processing (Nishad et al., 2017; Ziaee et al., 2019). Compared with the fluid, the powder has a longer shelf life, a less storage space, and is more convenient to use. Therefore, powdered model can facilitate practical application of model. The physical properties of standardized dietary model powder were discussed compared with commercial food powder (whole milk powder) as shown in Table 2.

**TABLE 2:** The physical characteristics of powdered standardized dietary model and commercial food powder (whole milk powder)

Parameters	Powdered model	Commercial powder
L^*^	94.16 ± 0.04^a^	87.12 ± 0.02^b^
a^*^	−0.27 ± 0.02^a^	0.75 ± 0.02^b^
b^*^	3.01 ± 0.05^a^	17.28 ± 0.04^b^
Moisture (%)	2.68 ± 0.02^a^	2.93 ± 0.04^b^
Repose angle (°)	37.28 ± 0.39^a^	36.46 ± 0.16^a^
Slide angle (°)	36.75 ± 0.83^a^	52.05 ± 1.46^b^
Bulk density (g/ml)	0.45 ± 0.02^a^	0.55 ± 0.01^b^

Note

Different letters in the table indicate significant difference (*p* < .05).

The flowability plays a critical role in food powder preparation and is inaccurate by determining using a single indicator. The repose and slide angle are common parameters of powder flowability. The smaller of repose angle and slide angle, the smaller of the friction, and the better the powder flowability. Table 2 shows that the repose angles of powdered standard meal and whole milk powder were not significantly different from whole milk powder (*p* > .05), however, as significantly decreased the moisture, slide angle and bulk density of powdered standard meal in comparison with whole milk powder (*p* < .05). Interestingly, in the powdered standardized diet model, L^*^ value was significantly higher than the whole milk powder (*p* < .05), however, as significantly decreased a^*^ and b^*^ value of powdered standard meal in comparison to whole milk powder (*p* < .05), which may be attributed to the different particle size or chemical composition of the different powder. These results indicated that the powdered standardized diet model has a moisture content, good flowability, and strong white appearance, which is helpful for practical production and application, which is similar to previous study (Zhang et al., 2019).

### 3.4 Characterization of reconstituted standardized dietary model

The reconstituted standardized dietary model properties (particle size, surface charge, and viscosity) were also measured and compared with the fresh samples as shown in Table 3. The particle size, Zeta potential, and viscosity of the reconstituted standardized dietary model were not significantly different from the fresh samples (*p* > .05), indicating the spray drying followed by reconstitution leads to a product similar to the fresh sample. In summary, the results from this study indicated that the model was satisfy: (a) easy to fabrication; (b) good stability; (c) small particle size and narrow particle size distribution; (d) strong practicability; (e) good reproducibility, which were similar to most physiological food state.

**TABLE 3:** The physical characteristics of fresh sample and reconstituted sample

Sample	Size/nm	Zeta potential/mV	Viscosity/Pa s
Fresh sample	295.59 ± 6.31^a^	−23.78 ± 0.15^a^	0.04 ± 0.00^a^
Reconstituted sample	310.75 ± 11.03^a^	−23.98 ± 0.22^a^	0.04 ± 0.01^a^

Note

Different letters in the table indicate significant difference (*p* < .05).

## 4 CONCLUSIONS

We prepared the O/W primary emulsions by different emulsification methods in order to provide the premise for preparing a stable and uniform standardized dietary model. Results demonstrated that the primary emulsions by high‐pressure homogenization in 12,000 psi had small particle size, narrow size distribution, great uniformity, and stability and should be stored at 4°C. We established then a standardized dietary model containing typical nutrients of protein, fat, carbohydrate, and mineral based on Chinese dietary reference intakes and Chinese dietary guidelines from the average for men and women of 18 years or older, which was dried into powder using spray drying compared with commercial food powder (whole milk powder) to facilitate the practical application of the standardized dietary model and then was reconstituted compared with the fresh sample. Results demonstrated that the model was satisfy :(a) easy to fabrication; (b) good stability; (c) small particle size and narrow particle size distribution; (d) strong practicability; (e) good reproducibility, which were similar to most physiological food state. This study warrants future work to explore the interactions between NPs and the model to better mimic the exposure of NPs in real life and to evaluate better the biological effect and toxicity of NPs via oral exposure. Moreover, the model may be used to study the biokinetics and bioavailability of food matrices after co‐exposure between NPs and the model, to evaluate the fate and transport of nano‐nutraceuticals, or to study enteric nutrient dynamics, bioavailability, and dissolution of ingested nutrients intake or drugs.

## CONFLICT OF INTEREST

The authors declare that they have no conflict of interest.

## ETHICAL APPROVAL

This study does not involve any human or animal testing.

## INFORMED CONSENT

Written informed consent was obtained from all study participants.
